# Tissue Inhibitor of Metalloproteinases-2 (TIMP-2) as a Prognostic Biomarker in Acute Kidney Injury: A Narrative Review

**DOI:** 10.3390/diagnostics14131350

**Published:** 2024-06-25

**Authors:** Charlotte Delrue, Marijn M. Speeckaert

**Affiliations:** 1 Department of Nephrology, Ghent University Hospital, 9000 Ghent, Belgium; charlotte.delrue@ugent.be; 2 Research Foundation-Flanders (FWO), 1000 Brussels, Belgium

**Keywords:** acute kidney injury, tissue inhibitor of metalloproteinases-2, matrix metalloproteinases

## Abstract

Acute kidney damage (AKI) is a serious and common consequence among critically unwell individuals. Traditional biomarkers, such as serum creatinine, frequently fail to detect AKI in its early stages, necessitating the development of new accurate early biomarkers. Tissue inhibitor of metalloproteinases 2 (TIMP-2) has emerged as a promising biomarker for predicting early AKI. The present narrative review investigates the role of TIMP-2 in AKI prediction in a variety of clinical scenarios. In the NephroCheck^®^ test, TIMP-2 exceeds established biomarkers for the early identification of AKI in terms of sensitivity and specificity when combined with insulin-like growth factor-binding protein 7 (IGFBP-7). Elevated levels of these biomarkers can provide a warning signal for AKI two to three days before clinical symptoms appear. TIMP-2 and IGFBP-7 have high predictive values, with an area under the curve (AUC) typically above 0.8, indicating good predictive capacity. For example, the [TIMP-2] × [IGFBP-7] product produced an AUC of 0.85 in surgical patients at high risk. In critically ill patients, a threshold of 0.3 (ng/mL)^2^/1000 demonstrated 92% sensitivity and 72% specificity. Elevated TIMP-2 levels have been correlated with higher mortality rates and the need for renal replacement therapy (RRT). In sepsis-associated AKI (SA-AKI), TIMP-2 levels combined with clinical prognostic models improved predictive accuracy (AUC: 0.822). Furthermore, elevated urine TIMP-2 levels were good predictors of AKI in pediatric patients after cardiac surgery, with AUC-ROC values of up to 0.848. Urine output and the presence of concomitant disorders may influence the prognostic accuracy of these biomarkers; therefore, more research is needed to fully understand their utility. The predictive value of TIMP-2 could be strengthened by combining it with other clinical parameters, reinforcing its role in the early detection and treatment of AKI.

## 1. Introduction

Acute kidney injury (AKI) is a prevalent clinical illness that has a major influence on patient outcomes. According to recent large-scale studies, globally, AKI affects over 13.3 million people annually, with low- and middle-income countries accounting for 85% of its cases [1]. It can afflict up to 50% of patients in intensive care units (ICUs), with an even higher frequency among those undergoing major surgery or suffering from severe sepsis [2]. Older age, pre-existing chronic kidney disease (CKD), diabetes mellitus, arterial hypertension, and heart failure are the main risk factors for AKI. The severity and duration of AKI have been associated with an increased risk of poor outcomes, including the development of CKD and eventually end-stage kidney disease (ESKD) and a higher mortality rate [3,4]. Long-term effects of AKI include cardiovascular diseases and increased healthcare expenses [5,6].

Over the last few decades, the development and application of biomarkers in AKI prognosis have advanced dramatically. Historically, serum creatinine and urine output were the key diagnostic diagnostics for AKI. However, these markers are unsatisfactory because they reflect kidney function rather than direct tissue damage, and they are late indicators of injury [7]. Proteomics and genomics developments have led to the discovery of several intriguing AKI biomarkers. The first studies concentrated on substances that respond to damage to kidney tissue, such as kidney injury molecule-1 (KIM-1), neutrophil gelatinase-associated lipocalin (NGAL), and interleukin-18 (IL-18) [8]. These biomarkers, in addition to being diagnostic, provide fundamental insights into the molecular mechanisms underlying kidney injury and repair [9]. Studies have demonstrated that increased levels of NGAL and KIM-1 can predict the severity and course of AKI [10]. Combining numerous biomarkers gives a holistic approach to AKI diagnosis and prognosis. For instance, the integration of NGAL, KIM-1, and IL-18 with clinical parameters enhances the prediction of adverse outcomes [11]. Novel biomarkers such tissue inhibitor of metalloproteinases-2 (TIMP-2) and insulin-like growth factor-binding protein-7 (IGFBP-7) are effective predictors of cell cycle arrest and improve prognosis [12]. TIMP-2 is one of the four members of the TIMP family, which includes peptidases known as matrix metalloproteinases (MMPs). It is an important regulator of extracellular matrix (ECM) turnover and plays a role in a variety of physiological and pathological processes, most notably kidney injury and repair [13,14,15,16,17]. IGFBP-7 is a member of the IGFBP family. It binds to insulin-like growth factors (IGFs) and modifies their function and bioavailability. In contrast to other IGFBPs, IGFBP-7 has a modest affinity for IGFs but is capable of performing a variety of IGF-independent activities. It is expressed in many different tissues, one of them being the kidneys, where it is crucial for cellular stress responses. It is well known that IGFBP-7 arrests the cell cycle in the G1 phase by increasing the synthesis of cyclin-dependent kinase inhibitors such as p21 and p27 [18].

The aim of the present review is to investigate the potential of TIMP-2 as a diagnostic target in AKI. It focusses on the functions of this protein and discusses the problems surrounding clinical application.

## 2. Tissue Inhibitor of Metalloproteinases-2

### 2.1. General Characteristics

TIMP-2 is a critical regulatory protein composed of 194 amino acids with a molecular weight of around 21 kDa. It has two different domains: N-terminal and C-terminal [19]. TIMP-2 has a dual regulatory function: it reduces MMP activity and facilitates pro-MMP-2 activation [20]. The N-terminal domain (the first 125 amino acids) is principally responsible for suppressing the enzymatic activity of active MMPs. This domain forms a reversible compound with the active site of MMPs in a 1:1 stoichiometry, effectively blocking the enzyme [19]. Molecular dynamics simulations and X-ray crystallography have shown that the N-terminal edge of TIMP-2 can adopt several conformations, potentially altering its binding affinity and specificity for distinct MMPs [21,22]. Although the C-terminal domain has received less attention, it is thought to play a role in modulating the activation of pro-MMP-2 [23,24,25]. TIMP-2 regulates MMP activity by forming a strong, noncovalent bond, particularly with MMP-2 (gelatinase A). Pro-MMP-2 can be activated on the cell surface by interacting with the hemopexin-like domain and the non-catalytic sections of membrane-type 1 matrix metalloproteinase (MT1-MMP), a process modulated by TIMP-2. This mechanism is essential for cell-mediated collagenolysis and tissue remodeling [16,23,24,25,26].

Although many different types of tissues have high expression levels of TIMP-2, it is mostly expressed in the glomeruli and tubular cells of the kidneys [27]. In kidney tissue, TIMP-2 is involved in the regulation of the ECM components, which is crucial for maintaining the structural and functional integrity of the kidney [28]. TIMP-2 expression is tightly regulated at the transcriptional and post-transcriptional stages. It is upregulated by cytokines and growth factors, such as transforming growth factor-beta (TGF-β), and is linked to fibrosis and kidney disease pathways [29,30]. TGF-β activates TIMP-2 expression via many signaling pathways, including small mothers against decapentaplegic (Smad) and mitogen-activated protein kinase (MAPK) [31]. The interplay of these pathways increases TIMP-2 transcriptional activity, which helps to regulate the ECM turnover [29]. In addition to TGF-β, several other cytokines and growth factors, including fibroblast growth factor (FGF) and epidermal growth factor (EGF), affect TIMP-2 production, but their activities are less well known [32].

### 2.2. Mechanism of Action

**Figure 1:**
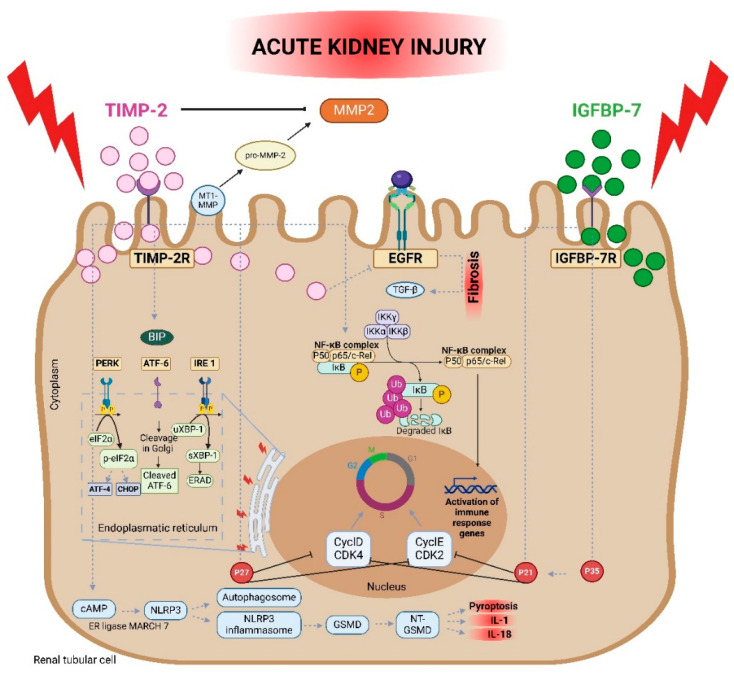
Schematic overview of the pathways affected by TIMP-2 in kidney damage. First of all, TIMP-2 has a dual effect on MMP2. It can directly inhibit the function of MMP2, but it can also stimulate MT1-MMP to form the pro-MMP2. Secondly, TIMP-2 and IGFBP-7 are both expressed in damaged tubular cells. They stimulate P27 and P21 and P35, respectively. This results in the inhibition of CyclD-CDK4 and CyclE-CDK2, causing an interruption of the G1 cell cycle of mitosis. Thirdly, EGFR signaling is affected by TIMP-2, by blocking EGFR. EGFR stimulation normally results in TGF-β-dependent kidney fibrosis, which is inhibited by TIMP-2. NF-κB is a key regulator in inflammatory responses. TIMP-2 stimulates the activation of immune response genes, causing an inflammatory response. Fourthly, TIMP-2 can trigger an ER stress response via interaction with BiP, an ER chaperone. This interaction triggers three endogenous ER stress sensors: PERK, ATF6, and IRE1. The PERK activation results in the phosphorylation of eIF2α, which causes the stimulation of ATF-4 and CHOP. ATF-4 increases the transcription of cytoprotective genes, whereas CHOP stimulates apoptosis. ATF-6 results in the production of more ER chaperones, and the IRE1 pathway activates XBP-1, which in turn produces the expression and synthesis of ER chaperones and ER-associated degradation proteins. Lastly, the cAMP-stimulated activation of NLRP3 results in the formation of an autophagosome and NLRP3 inflammasome, which stimulates GSMD resulting in increased pyroptosis and inflammatory markers such as IL-1 and IL-18. Abbreviations: TIMP-2: tissue inhibitor of metalloproteinases-2; MMP2: matrix metalloproteinase-2; MT1-MMP: membrane type-1 matrix metalloproteinase; pro-MMP2: pro-matrix metalloproteinase-2; IGFBP-7: insulin-like growth factor-binding protein 7; CyclD-CDK4: cyclin-dependent protein kinase complexes 4; CyclE-CDK2: cyclin-dependent protein kinase complexes 2; EGFR: epidermal growth factor receptor; TGF-β: transforming growth factor-beta; NF-κB: nuclear factor kappa-light-chain-enhancer of activated B cells; ER: endoplasmatic reticulum; BIP: binding immunoglobulin protein; PERK: protein kinase RNA-like ER kinase; ATF-6: activating transcription factor-6α; IRE1: inositol-requiring enzyme 1; eIF2α: eukaryotic initiation factor 2 alpha; ATF-4: activating transcription factor 4; CHOP: C/EBP-homologous protein; XBP-1: activates X box-binding protein 1; cAMP: cyclic adenosine monophosphate; NLRP3: NOD-like receptor protein 3; GSMD: gasdermin D; NT-GSMD: N-terminal gasdermin D; and IL: interleukin.

TIMP-2 is an important molecule that regulates the turnover of the ECM and is required for inflammation and numerous cellular activities, including cell differentiation, cell death, and cell proliferation, especially in the context of kidney injury. The most widely recognized role of TIMP-2 is its ability to inhibit MMP activity, particularly MMP-2 (gelatinase A) (Figure 1). TIMP-2 directly inhibits the active site of MMP-2, which is involved in the breakdown of collagen and gelatin, both of which are essential components of the ECM. This inhibition is critical for avoiding fibrosis and managing the ECM remodeling during tissue repair. Furthermore, TIMP-2 contributes to the activation of pro-MMP-2. This is accomplished by interacting with MT1-MMP, which causes the cellular activation of pro-MMP-2. This dual involvement shows the complex modulation of TIMP-2 in the ECM dynamics [33]. During AKI, damaged tubular cells express both TIMP-2 and IGFBP-7, which act complementarily. TIMP-2 primarily regulates the ECM turnover and inhibits MMP-2, thereby maintaining tissue homeostasis and preventing kidney fibrosis. To recover from injury and maintain structural and functional integrity, the kidneys require the precise alteration of the ECM [13,14,15,16,17]. IGFBP-7, on the other hand, promotes the expression of p21 and p27, leading to cell cycle arrest. This combined action helps to prevent the proliferation of damaged cells and allows for repair, thus offering a powerful predictive tool for AKI [34].

TIMP-2 conducts other biological functions in addition to controlling the ECM. One of its primary activities is to block angiogenesis, which is a necessary mechanism for wound healing and tumor growth. The anti-angiogenic activity of TIMP-2 stems from both its direct impact on endothelial cell proliferation and its ability to inhibit MMPs involved in vascular remodeling [35]. TIMP-2 prevents MMP-2 from being activated, which is necessary for angiogenesis and the degradation of the ECM. This inhibition stops the basement membrane from rupturing and new blood vessel formation [36]. TIMP-2 also interacts with endothelial cell surface integrins to modify their signaling pathways and decrease migration and proliferation of endothelial cells. TIMP-2 binds to integrin α_3_β_1_ of endothelial cells, interfering with downstream signaling pathways leading to, e.g., fibroblast growth factor-2 (FGF-2)-induced p42/44 (MAPK) activation [37].

TIMP-2 is also involved in the regulation of the epidermal growth factor receptor (EGFR) pathways, which is essential for cell survival and proliferation. In cases of AKI, chronic EGFR activation has been linked to kidney fibrosis, which is dependent on transforming growth factor-β (TGF-β). By blocking EGFR activation, TIMP-2 curtails EGF-mediated mitogenic signaling affecting how renal tubular cells respond to cellular damage [38,39].

TIMP-2 downregulation seems to affect the nuclear factor kappa-light-chain-enhancer of activated B cell (NF-κB) pathway resulting in altered apoptosis and inflammation. In cultured kidney cells, TIMP-2 knockdown reduced lipopolysaccharide (LPS)-induced cytokine production, apoptosis, and cell damage [40]. This inducible dimeric transcription factor, NF-κB, is made up of the NF-κB1 (p50) and RelA (p65) subunits. When NF-κB is activated, it becomes freed from its inhibitor, IκB, and moves from the cytoplasm to the nucleus, where it attaches to target gene promoters. TIMP-2 phosphorylates both IκB and NF-κB, increases transcriptional NF-κB activity, and raises IL-8 levels in TIMP-2-overexpressed cells, among other impacts on the NF-κB pathway [41].

TIMP-2 is also implicated in endoplasmic reticulum (ER) stress, which has been connected to a variety of pathological diseases, including kidney disease. In sepsis-associated acute kidney injury (SA-AKI), TIMP-2 enhances ER stress-induced apoptosis. TIMP-2 interacts with binding immunoglobulin protein (BiP), an ER chaperone, allowing for the extracellular secretion of TIMP-2 and thereby inducing ER stress. This pathway demonstrates an unique mechanism by which TIMP-2 leads to kidney damage [42]. When endogenous ER stress is generated, the ER chaperone BiP attaches itself to unfolded proteins and triggers three endogenous ER stress sensors: protein kinase RNA-like ER kinase (PERK), activating transcription factor 6α (ATF6), and inositol-requiring enzyme 1 (IRE1). By phosphorylating eIF2α, activated PERK reduces the translation of proteins. Additionally, phosphorylated eIF2α stimulates ATF4 transcription, which in turn increases the transcription of cytoprotective genes. Apoptosis is induced by phosphorylated eIF2α via the overexpression of C/EBP homologous protein (CHOP). ATF6 relocates to the Golgi apparatus and is cleaved by proteases to become an active transcription factor. ER chaperones are stimulated to express when ATF6 is activated. The activation of the endoribonuclease activity of IRE1, which is triggered by ER stress, also leads to the synthesis of spliced and active X box-binding protein 1 (XBP-1s), which in turn produces the expression and synthesis of ER chaperones and ER-associated degradation proteins [43].

A mouse model was used to evaluate the effect of kidney tubule-specific TIMP-2 deletion on kidney damage and inflammation. Timp2-knockout animals had more severe kidney injury than wild-type mice, as demonstrated by higher levels of pyroptosis markers such as NOD-like receptor protein 3 (NLRP3), caspase-1, and gasdermin D (GSDMD) during the early stages of SA-AKI. In contrast, when exogenous TIMP-2 was administered to Timp2-knockout mice, they were significantly protected against kidney injury and inflammation. In vitro tests using recombinant TIMP-2 protein showed that the knockdown of TIMP-2 increased pyroptosis in renal tubular cells that were stimulated with LPS. Moreover, the external addition of TIMP-2 markedly increased the ubiquitination and subsequent breakdown of NLRP3 through autophagy. Increasing levels of cyclic adenosine monophosphate (cAMP) inside the cells achieved this outcome. The participation of the E3 ligase membrane-associated ring finger (C3HC4) 7 (MARCH7) was required for the cAMP-mediated process of degrading NLRP3. As a result, this decreased the occurrence of pyroptosis in downstream cells and eased the initial harm to tubular cells. These results emphasize the protective effect of extracellular TIMP-2 in SA-AKI by diminishing tubular pyroptosis [44].

## 3. Analytical Aspects

TIMP-2 can be found and measured using a variety of techniques, including enzyme-linked immunosorbent assays (ELISAs), zymography, reverse transcription polymerase chain reaction (RT-PCR), and surface plasmon resonance (SPR). These methods differ in terms of their practical utility, sensitivity, and specificity. ELISA is a commonly employed method for TIMP-2 identification with the use of TIMP-2-specific monoclonal or polyclonal antibodies. Both the capture and detection antibodies can target different TIMP-2 epitopes in a one-step sandwich-style experiment. The use of monoclonal antibodies that target specific epitopes allows ELISA to detect TIMP-2 at as low as 0.5 pM with good specificity [45]. The linear detection range of the test is 6.3–50 µg/L. Typically, the intra- and inter-assay coefficients of variation (CV) are less than 10%, demonstrating high reproducibility and precision.

Zymography is another method for detecting TIMP-2. This technique involves separating proteins using gel electrophoresis and then detecting protease activity. Zymography is very effective for examining the interaction between TIMP-2 and its target proteases, such as MMP-2, since it allows us to see the suppression of enzyme activity. This approach, while less sensitive than ELISA, yields qualitative data on TIMP-2 activity [46].

TIMP-2 mRNA levels are quantified using reverse transcription polymerase chain reaction (RT-PCR), which provides insights into gene expression patterns. This approach is extremely sensitive and can detect low quantities of mRNA, allowing for the investigation of TIMP-2 expression in a variety of tissues and circumstances. RT-PCR is particularly useful in research settings where understanding the regulation of TIMP-2 expression is critical [47].

Surface plasmon resonance (SPR) is a label-free detection method that measures the binding interactions between molecules in real-time. SPR has been used to study the interaction between TIMP-2 and MMP-2, providing detailed kinetic data on the binding affinity and dissociation rates. The sensitivity of SPR can be enhanced using gold nanoparticles, which allow for the detection of TIMP-2 at concentrations as low as 0.5 pM [48].

NephroCheck^®^ (BioMérieux, Marcy-l’Étoile, France) is a commercial product combining two urinary biomarkers, TIMP-2 and IGFBP-7, to assess the risk of AKI. The NephroCheck^®^ test measures the concentrations of these biomarkers in urine, indicating cell cycle arrest, a response to early kidney stress before significant damage occurs. The test has demonstrated a good sensitivity and specificity in predicting AKI development, with an area under the curve (AUC) for risk prediction often exceeding 0.8, indicating good predictive power [49]. The intra-assay and inter-assay CVs for NephroCheck^®^ are typically low, showing high reproducibility.

Several factors can influence the accuracy and reliability of TIMP-2 detection. These include the quality and specificity of antibodies used in assays, the purity of samples, and the presence of interfering substances that may affect binding interactions. Standardization of protocols and rigorous validation of assays are essential to ensure consistent and reliable results [50]. Chindarkar et al. (2016) conducted an extensive examination into the reference intervals for the biomarkers TIMP-2 and IGFBP-7 in both healthy individuals (*n* = 378) and those with persistent chronic diseases such as diabetes mellitus, arterial hypertension, or chronic heart disease, but no AKI. The primary purpose of this study was to establish baseline levels for these biomarkers, as this is critical to their clinical relevance in diagnosing and forecasting AKI. The reference intervals for healthy people and those with stable chronic illnesses did not differ statistically in any way, suggesting that persistent chronic morbidities did not raise the baseline values of these biomarkers [51]. Other research groups reported that co-morbid illnesses such diabetes mellitus, cardiovascular disease, and chronic kidney disease (CKD) can affect TIMP-2 levels. Baseline elevations in TIMP-2 caused by these diseases may conceal acute alterations associated with kidney damage. It can be difficult to differentiate between acute and chronic kidney stresses in individuals with CKD due to the possibility of persistently high TIMP-2 levels [50].

## 4. Clinical Evidence on TIMP-2

Numerous studies have proven the usefulness of TIMP-2 as a biomarker for AKI, highlighting its diagnostic accuracy and potential to improve patient outcomes.

### 4.1. Preclinical Studies

The possible significance of urine TIMP-2 and IGFBP-7 has been examined in an SA-AKI rat model. Urine TIMP-2 and IGFBP-7 concentrations were substantially higher in rats with moderate-to-severe AKI than in rats without AKI. A strong predictive accuracy was demonstrated by the product [TIMP-2] × [IGFBP-7], with an area under the curve (AUC) of 0.89 [95% confidence interval (CI): 0.80–0.98] compared to the AUC of 0.78 for plasma cystatin C. [TIMP-2] × [IGFBP-7] levels have predictive value for mortality (AUC: 0.69, 95% CI: 0.53–0.85), suggesting that they have broader prognostic use in AKI caused by sepsis [52]. The potential of TIMP-2 as a biomarker for SA-AKI was subsequently studied in three groups of 56 male rats (6 controls, 24 sham-operated rats, and 24 rats suffering from sepsis). Plasma TIMP-2 levels rose 6 h after surgery and stayed elevated over time. Immunohistochemical examination of the kidneys revealed that TIMP-2 was continuously expressed in the sepsis-induced group throughout the trial. The group of rats with sepsis showed higher expression of TIMP-2 and pyroptosis-related proteins (NLRP3, IL-1β, caspase-1, and GSDMD) compared to the sham-operated group, suggesting pyroptosis plays a role in AKI [53].

### 4.2. Clinical Studies

#### 4.2.1. All-Cause Acute Kidney Injury

A meta-analysis looked at the prognostic value of urine TIMP-2 and IGFBP-7 for all-cause AKI. Twenty trials, totaling 3625 patients, were included. The pooled sensitivity of urine [TIMP-2] × [IGFBP-7] for AKI diagnosis was 0.79 (95% CI: 0.72–0.84) and specificity was 0.70 (95% CI: 0.62–0.76). The positive likelihood ratio was 2.6 (95% CI: 2.1–3.3), the negative likelihood ratio was 0.31 (95% CI: 0.23–0.40), and the diagnostic odds ratio was 8 (95% CI: 6–13). The AUC was 0.81 (95% CI: 0.78–0.84), indicating good diagnostic accuracy. Subgroup analyses showed variability based on AKI severity, timing of biomarker measurement, and clinical settings [54]. The predictive value of urine [TIMP-2] × [IGFBP-7] levels for unfavorable outcomes in patients with AKI has been examined in another meta-analysis, including 10 prospective trials (*n* = 1559 AKI participants). The [TIMP-2] × [IGFBP-7] levels were measured using the NephroCheck^®^ test in nine studies and ELISA kits in one study. The endpoints included non-recovery of kidney function, the requirement for renal replacement therapy (RRT), mortality, and stage 3 AKI. The pooled sensitivity was 0.82 (95% CI: 0.77–0.86), specificity was 0.64 (95% CI: 0.61–0.67), diagnostic odds ratio was 14.06 (95% CI: 7.31–27.05), positive likelihood ratio was 2.859 (95% CI: 2.15–3.77), and negative likelihood ratio was 0.28 (95% CI: 0.20–0.40). The Q* value was 0.7970 (standard error: 0.0299), and the AUC was 0.8864 (standard error: 0.0306). The results of the investigation showed that urine [TIMP-2] × [IGFBP-7] is a valid biomarker for predicting a worse outcome in patients with AKI. Many studies found that the biomarker had fair specificity and great sensitivity, although there was a lot of heterogeneity, owing to differences in trial aims and cutoff values. Sensitivity analysis demonstrated that the Titeca-Beauport et al. study [55] contributed to the significant heterogeneity. The heterogeneity in the pooled diagnostic odds ratio and sensitivity decreased after this trial was excluded. Comparing the RRT/death subgroup to the non-recovery of kidney function/stage 3 AKI subgroup, subgroup analysis showed that [TIMP-2] × [IGFBP-7] had a better predictive value [56]. A meta-analysis involving four trials (*n* = 382) was conducted to evaluate the predictive power of TIMP-2 and IGFBP-7 for chronic AKI. The prediction of chronic AKI had a pooled specificity of 0.68 (95% CI: 0.50–0.82) and a pooled sensitivity of 0.61 (95% CI: 0.46–0.75) for [TIMP-2] × [IGFBP-7]. The AUC was 0.69 (95% CI: 0.65–0.73). Although [TIMP-2] × [IGFBP-7] is not a perfect predictor, it nevertheless has great relevance with reasonable sensitivity and specificity in early detection, especially when combined with other clinical assessments and biomarkers [57] (Table 1).

#### 4.2.2. Acute Kidney Injury after Surgery

The potential of urinary TIMP-2 and IGFBP-7 to predict AKI in high-risk surgical patients was assessed in 107 surgical patients. Patients with AKI showed significantly higher levels of [TIMP-2] × [IGFBP-7] compared to those who did not. In particular, the combination of these biomarkers produced a good predictive value with an AUC of 0.85. Traditional risk indicators and clinical markers frequently fail to predict AKI with high accuracy. Adding [TIMP-2] × [IGFBP-7] in multivariable models significantly improved the predictive power. The indicators accurately differentiated between patients who were at a higher risk of having AKI and those who were not, with a high degree of sensitivity (92%) and specificity (88%). Patients having hepatic, transplant, and septic procedures had the highest median values of [TIMP-2] × [IGFBP-7] of 1.24, 0.45, and 0.47 (ng/mL)^2^/1000, respectively. This fluctuation emphasizes the reliability of [TIMP-2] × [IGFBP-7] as a biomarker for different surgical stresses and patient situations. The [TIMP-2] × [IGFBP-7] test was the strongest predictor of AKI in multivariable models (*p* < 0.001), exceeding other perioperative risk indicators [50]. The predictive value of TIMP-2 and IGFBP-7 for postoperative AKI has also been investigated in prospective, single-site observational trial with 93 adult patients undergoing abdominal aortic operations. Median [TIMP-2] × [IGFBP-7] levels were slightly higher in patients who developed AKI compared to those who did not. On the first postoperative day, the median levels were 0.39 (ng/mL)^2^/1000 [interquartile range (IQR): 0.13–1.05] in patients with AKI compared to 0.23 (IQR: 0.14–0.53) in patients without AKI. However, the difference was not statistically significant. Similarly, the immediate postoperative levels were also comparable between the two groups [0.2 (IQR: 0.08–0.42) vs. 0.18 (IQR: 0.09–0.46)]. At the 0.3 (ng/mL)^2^/1000 cutoff, the sensitivity was 58%, and the specificity was 58%, indicating a moderate ability to correctly identify patients at risk of AKI and to exclude those not at risk. At the higher cutoff of 2.0 (ng/mL)^2^/1000, the sensitivity dropped to 16%, while the specificity increased to 98%, showing that this threshold was highly specific but poorly sensitive. In this particular surgical setting, the biomarkers were unable to accurately differentiate between patients who would develop AKI and those who would not [65].

#### 4.2.3. Septic-Associated Acute Kidney Injury

In a prospective observational study of 198 people with SA-AKI, the combination of urine TIMP-2 and IGFBP-7 at baseline had a modest predictive value for non-recovery of kidney function, with an AUC of 0.782. When the [TIMP-2] × [IGFBP-7] biomarker levels were integrated with the clinical prognostic model, the predictive accuracy improved, resulting in an AUC of 0.822. This combined model achieved a sensitivity of 88.3% and a specificity of 59.5% [66]. The PHENAKI study is a post hoc analysis of data from the multicenter, prospective AKI-CHECK trial, which included 184 patients who developed septic shock and SA-AKI within 6 h of beginning catecholamine treatment. The study sought to determine whether the serial assessments of urine TIMP-2 and IGFBP-7 levels, together with traditional kidney function indicators within the first 24 h, might detect distinct SA-AKI subphenotypes. Three unique SA-AKI subphenotypes were found using hierarchical principal component analysis. Subphenotype A had normal urine output, low serum creatinine, and low [TIMP-2] × [IGFBP-7] levels. These patients generally had less severe illness and a higher likelihood of rapid kidney function recovery. Only one patient required RRT, and kidney function normalized in 54% of patients within 24 h. Subphenotype B comprised patients with pre-existing CKD, higher serum creatinine, low urine output, and intermediate [TIMP-2] × [IGFBP-7] levels. This group had a higher prevalence of CKD and poorer kidney function at baseline. Only 9.4% of patients experienced normal kidney function, whereas 19% required RRT. Subphenotype C was identified by reduced urine production, high [TIMP-2] × [IGFBP-7] levels, and intermediate serum creatinine. These individuals had more severe illness, as evidenced by higher lactate levels and non-renal SOFA scores. A large number of these individuals developed stage 3 AKI and required RRT. The 7-day RRT-free survival rates varied significantly between the subphenotypes: 91% for subphenotype A, 50% for subphenotype B, and 27% for subphenotype C. After accounting for covariates, the 7-day RRT-free survival rates were 85%, 59%, and 56%, respectively. Patients with subphenotype C, with their very high [TIMP-2] × [IGFBP-7] levels and low urine output, represent a high-risk group needing close monitoring and possibly aggressive interventions. The integration of [TIMP-2] × [IGFBP-7] with conventional kidney function variables significantly improved the classification of SA-AKI subphenotypes, allowing for the better stratification of patients in terms of their risk for progression and mortality [67].

#### 4.2.4. Acute Kidney Injury after Cardiac Arrest and after Cardiac Surgery

Titeca-Beauport et al. (2019) evaluated the efficacy of the biomarkers TIMP-2 and IGFBP-7 in identifying patients at risk of severe AKI after cardiac arrest. The study aimed to see if measuring urine [TIMP-2] × [IGFBP-7] levels immediately after cardiac arrest might reliably predict the onset of severe AKI (KDIGO stage 3) within 48 h of ICU admission. The major outcome was the development of severe AKI, which typically requires RRT and is linked with increased morbidity and mortality. Urine samples were taken from 115 participants in the study, about 240 min after cardiac arrest. Of the 32 patients (28%) who suffered severe AKI, 11 required RRT. Patients with severe AKI showed considerably higher baseline levels of [TIMP-2] × [IGFBP-7] than those without AKI [1.57 (0.80–6.62) vs. 0.17 (0.05–0.59) (ng/mL)^2^/1000, *p* < 0.001]. This considerable discrepancy highlights the potential of the biomarkers as early indicators of serious kidney failure. [TIMP-2] × [IGFBP-7] had strong discriminative capacity, with an AUC of 0.91. The best threshold value with 97% sensitivity and 72% specificity was found to be 0.39 (ng/mL)^2^/1000. Additionally, the study contrasted [TIMP-2] × [IGFBP-7] with other conventional indicators of kidney function, such as urine output and serum creatinine. With an AUC of 0.73 (*p* = 0.005), baseline [TIMP-2] × [IGFBP-7] was considerably more predictive of severe AKI than baseline serum creatinine. It showed a small but not statistically significant increase over the baseline urine output. The predictive performance of [TIMP-2] × [IGFBP-7] further improved when paired with serum creatinine and urine output, indicating the additional utility of these biomarkers in clinical risk assessment [55].

Research on the prognostic efficacy of urine [TIMP-2] × [IGFBP-7] for postoperative AKI in patients who have undergone cardiac surgery has yielded conflicting results. In a prospective cohort trial of 557 adult patients undergoing cardiac surgery, urinary TIMP-2 combined with IGFBP-7 was an excellent early indication of cardiac surgery-associated acute kidney injury (CSA-AKI) and related short-term negative outcomes. AKI was detected in 24.06% of the 557 individuals, with 5.9% experiencing moderate-to-severe AKI. There was an association between diabetes mellitus, higher baseline blood creatinine levels, and age with an increased risk of AKI. Patients who developed AKI had significantly higher scores on the simplified renal index, EuroSCORE II, and Cleveland Clinic Score when compared to those without AKI. Patients with AKI had significantly higher urinary [TIMP-2] × [IGFBP-7] levels, with an AUC of 0.66 for predicting all AKI and 0.70 for predicting stages 2 and 3. A calculated threshold [TIMP-2] × [IGFBP-7] (NephroCheck^®^) value of 0.265 (ng/mL)^2^/1000 demonstrated sensitivity and specificity of 44.0% and 83.9%, respectively. The study confirms that urinary [TIMP-2] × [IGFBP-7] levels are predictive of CSA-AKI and can aid in identifying patients at risk for short-term adverse outcomes. Despite the positive results, clinical outcomes vary, and other factors such as prior health problems and concurrent illnesses can have an impact on diagnosis accuracy. Combining [TIMP-2] × [IGFBP-7] with other clinical risk scores, such as SRI or EuroSCORE II, improves its predictive performance [58]. In another trial, nine percent of the 230 patients who had undergone cardiac surgery developed AKI. [TIMP-2] × [IGFBP-7] baseline values were normal, although individuals with AKI had higher levels than patients without it. The receiver operating characteristic (ROC) analysis showed an AUC of 0.78 for [TIMP-2] × [IGFBP-7], with the best cutoff level at 2.0 mg/L (sensitivity 83.9%, specificity 73.8%). Higher [TIMP-2] × [IGFBP-7] levels correlated with AKI severity and the necessity for RRT, with patients showing levels > 2 mg/L being at significantly higher risk for severe AKI and requiring RRT [59].

A thorough meta-analysis (8 studies, *n* = 552 patients) assessed the predictive efficacy of urine [TIMP-2] × [IGFBP-7] for CSA-AKI. Urine [TIMP-2] × [IGFBP-7] had a combined sensitivity of 0.79 (95% CI: 0.71–0.86) and specificity of 0.76 (95% CI: 0.72–0.80). The pooled likelihood ratios for good and negative outcomes were 3.49 (95% CI: 2.44–5.00) and 0.31 (95% CI: 0.19–0.51), respectively. The diagnostic odds ratio for [TIMP-2] × [IGFBP-7] showed outstanding overall performance, with a combined sensitivity and specificity of 14.89 (95% CI: 7.31–30.32). The AUC was 0.868, with a Q* value of 0.799, confirming the high diagnostic accuracy. The threshold for identifying AKI was one important aspect that was found to be responsible for some of the variability in diagnostic performance. Sensitivity analysis revealed that the robustness of the results was reinforced when studies with noticeably different patient populations or techniques were excluded with no effect on the overall results [68].

In contrast to the previous studies, other trials found that these biomarkers were insufficient to predict postoperative AKI, highlighting the need for more research to fully comprehend the variability of their performance in other situations. In a prospective observational study on 108 consecutive patients undergoing open heart surgery in a single center, urinary [TIMP-2] × [IGFBP-7] did not show significant predictive power for postoperative AKI. The biomarkers tested right after cardiopulmonary bypass showed a sensitivity of only 13% and a specificity of 82% for predicting AKI at a threshold of >0.3 (ng/mL)^2^/1000. The sensitivity rose to 47% on the first postoperative day, but the specificity decreased to 59%. The sensitivity of the test was poor, and its specificity was intermediate, making it challenging to accurately identify individuals who will develop AKI. The study found no statistically significant difference in [TIMP-2] × [IGFBP-7] levels between patients who experienced surgical AKI and those who did not at any of the examined time points. Potential contributing factors include differences in patient demographics, the type and extent of heart surgery performed, perioperative care methods, and underlying comorbidities. These variables may have an impact on biomarker levels and prediction accuracy, necessitating a more tailored approach to their application in different clinical contexts [60].

#### 4.2.5. Acute Kidney Injury in Critically Ill Patients

The therapeutic effectiveness of urine TIMP-2 and IGFBP-7 for the risk stratification of AKI in critically ill patients was examined in a large prospective multicenter trial. The objective was to evaluate the efficacy of [TIMP-2] × [IGFBP-7] in identifying individuals at high risk for AKI, with a high sensitivity cutoff of >0.3 (ng/mL)^2^/1000. The study included 420 critically sick patients from various ICUs, ensuring a representative sample of patients in real-world clinical settings. The objective was to predict moderate-to-severe AKI within 12 h of assessment using the urine [TIMP-2] × [IGFBP-7] biomarker.

The sensitivity of the urinary [TIMP-2] × [IGFBP-7] test at the prespecified cutoff of 0.3 (ng/mL)^2^/1000 was 92% (95% CI: 85–98), with a negative likelihood ratio of 0.18 (95% CI: 0.06–0.33), indicating high predictive accuracy for imminent AKI. Critically sick individuals with urinary [TIMP-2] × [IGFBP-7] levels > 0.3 (ng/mL)^2^/1000 had a sevenfold higher chance of developing AKI (95% CI: 4–22) compared to those with lower levels. In multivariate models that included clinical variables alone, the AUC was 0.70 (95% CI: 0.63–0.76). When urinary [TIMP-2] × [IGFBP-7] was added to these models, the AUC improved significantly to 0.86 (95% CI: 0.80–0.90), highlighting the substantial enhancement in predictive power offered by these biomarkers. Higher biomarker levels were linked to an increased chance of developing AKI, as well as poorer overall outcomes, such as higher likelihood of needing RRT and higher mortality rate [61].

In a small study of 98 patients admitted to the ICU, 53 (54.1%) had stage 2 or 3 AKI, with just 23 (23.5%) meeting the creatinine criterion alone. Patients with stage 2 or 3 AKI had significantly higher median Nephrocheck^®^ values than patients with stage 1 or no AKI [0.97 (0.48–1.99) vs. 0.46 (0.22–1.17), *p* = 0.005]. However, the AUC-ROC for Nephrocheck^®^ was 0.66 (95% CI: 0.56–0.77), indicating limited predictive performance. Nephrocheck^®^ values were inversely correlated with urine output (ρ = −0.46, *p* < 0.001) at the time of measurement. Multivariable logistic regression analysis showed Nephrocheck^®^ was not independently associated with stage 2 or 3 AKI [OR: 1.06 (95% CI: 0.74–1.53)], whereas urine output was a significant predictor [OR: 0.98 per mL/h increase (95% CI: 0.97–1.00), *p* = 0.007]. When urine output was taken into account, Nephrocheck^®^ did not predict AKI independently, emphasizing the need of adding urine output in AKI risk assessments [69].

#### 4.2.6. Acute Kidney Injury in Children

Urine TIMP-2 is a promising early biomarker for AKI in critically ill children, enabling earlier diagnosis and treatment as compared to conventional methods. In a prospective cohort trial comprising forty-two severely unwell neonates, 13% of the children experienced AKI. Increased urine TIMP-2 levels were reliably linked to AKI, even before conventional indicators indicated the impairment of the kidneys. Patients with AKI had significantly higher urinary TIMP-2 levels from day 1, which preceded increases in serum creatinine and decreases in urine output on days 3 and 5, respectively. TIMP-2 has the potential to be an early biomarker, as evidenced by the substantial connection that was discovered between day 1 TIMP-2 levels and day 3 serum creatinine levels [70].

Urinary TIMP-2 and IGFBP-7 are also useful early indicators for diagnosing AKI in pediatric patients following heart surgery. These biomarkers, especially when used in conjunction as NephroCheck^®^, provide a reliable way of early detection, which is critical for minimizing AKI progression and improving patient outcomes. Out of 101 patients, 62.4% experienced AKI stage ≥1 within 48 h and 30.7% within 12 h. TIMP-2 and IGFBP-7 were also significant predictors of AKI after adjusting for urine dilution. For AKI stages ≥1 and ≥2, TIMP-2 had AUC-ROC values of 0.778 and 0.830. The AUC-ROC value for IGFBP-7 was 0.834 for AKI stage ≥2 and 0.796 for AKI stage ≥1. The combined NephroCheck^®^ test showed high predictive performance, with AUC-ROC values of 0.734 and 0.774 for AKI stages ≥1 and ≥2, respectively. NGAL and chitinase 3 Like 1 (CHI3L1) also predicted AKI but with lower AUC-ROC values compared to TIMP-2 and IGFBP-7 [71].

Finally, urinary TIMP-2 and IGFBP-7 are valuable biomarkers for the early detection of AKI in neonates after congenital heart surgery requiring cardiopulmonary bypass (CPB). In a small study of 36 neonates, 19 patients (53%) developed AKI: 13 with stage 1, 5 with stage 2, and 1 with stage 3. No patients required RRT. Urinary [TIMP-2] × [IGFBP-7] levels were significantly higher in patients with AKI at 24 h post-CPB [1.1 vs. 0.27 (ng/mL)^2^/1000; *p* = 0.0019]. The ROC curve for [TIMP-2] × [IGFBP-7] at 24 h post-CPB showed an AUC of 0.848, indicating high sensitivity and specificity for predicting AKI [72].

In contrast to the studies above, the combination of TIMP-2 and IGFBP-7 performed poorly to modestly in diagnosing cisplatin-induced AKI in juvenile patients [62].

#### 4.2.7. Acute Kidney Injury in Geriatrics

In a prospective observational cohort study, the relationship between urinary TIMP-2 and IGFBP-7 levels and renal non-recovery in 209 critically ill geriatric patients with AKI was studied. Kidney function was restored in 117 (56.0%) patients, but not in the other 92 patients (44.0%). Patients with renal non-recovery had higher APACHE II scores, more severe AKI (stages 2–3), and higher [TIMP-2] × [IGFBP-7] levels compared to those who recovered. The regression model incorporating [TIMP-2] × [IGFBP-7] demonstrated a fair predictive value for renal non-recovery (AUC: 0.774, *p* < 0.001), with an optimal threshold of 0.81 (ng/mL)^2^/1000. Combining [TIMP-2] × [IGFBP-7] with AKI severity and APACHE II score improved the predictive accuracy (AUC: 0.851) [73].

#### 4.2.8. Acute Kidney Injury and COVID-19

The efficacy of urine TIMP-2 and IGFBP-7 to predict AKI in patients with COVID-19 has been examined. A multicenter, prospective, observational study was conducted at twelve centers across Europe and the United Kingdom. It included adult patients with mild-to-severe COVID-19-related acute respiratory distress syndrome (ARDS). Urinary TIMP-2 and IGFBP-7 levels were measured at the start of the study and 12 and 24 h later. The primary outcome was the development of moderate or severe AKI within seven days, as defined by the KDIGO criteria. Among 300 patients, 39 (13%) developed moderate or severe AKI within seven days. At enrollment, urinary [TIMP-2] × [IGFBP-7] had a high predictive value for AKI, with an AUC-ROC of 0.89 (95% CI: 0.84–0.93). The biomarker levels were significantly higher in patients who developed AKI both at enrollment and 12 h post-enrollment. The AUC-ROC for [TIMP-2] × [IGFBP-7] was 0.67 (95% CI: 0.56–0.78) at 12 h and 0.58 (95% CI: 0.46–0.70) at 24 h post-inclusion, demonstrating that the predictive value was strongest during enrollment. Adding [TIMP-2] × [IGFBP-7] to clinical models enhanced risk prediction for AKI, outperforming clinical factors alone [74]. When measuring urinary biomarkers at 12 h after ICU admission, patients with severe AKI had higher [TIMP-2] × [IGFBP-7] levels [63]. In critically ill patients with COVID-19, the combination of [TIMP-2] × [IGFBP-7] and clinical data proved helpful in identifying subclinical AKI [75].

#### 4.2.9. Drug- and Contrast-Induced Acute Kidney Injury

Subtle variations in TIMP-2 and IGFBP-7 might predict drug-induced AKI. A prospective study (21 patients with AKI and 28 non-AKI matched controls) was conducted with serial urine collections from patients treated with nephrotoxic drugs, including vancomycin, aminoglycosides, amphotericin, foscarnet, or calcineurin inhibitors. Significantly higher absolute, normalized, and composite levels of TIMP-2 and IGFBP-7 were observed in AKI cases compared to controls as early as 2–3 days before AKI onset (all *p* < 0.05). More than 70% of patients with biomarker levels above the 75th percentile developed AKI. Normalized TIMP-2 levels 2–3 days before AKI had the highest predictive accuracy with an AUC-ROC of 0.81, followed by composite [TIMP-2] × [IGFBP-7] with an AUC-ROC of 0.78. The composite biomarker value [TIMP-2] × [IGFBP-7] > 0.01 (ng/mL)^2^/1000 predicted AKI with a sensitivity of 79% and a specificity of 60% [76].

In the secondary analysis of the Prevention of Serious Adverse Events following the Angiography (PRESERVE) trial, the prediction of cardiovascular events by TIMP-2 and IGFBP-7 was investigated. Among the 922 participants, 119 (12.9%) developed cardiovascular events, and 73 (7.9%) developed contrast-associated acute kidney injury (CA-AKI), with the majority (90%) being stage 1 AKI. The percentage of individuals with CA-AKI and urinary [TIMP-2] × [IGFBP-7] concentrations did not significantly differ between those who experienced cardiovascular events and those who did not [77]. Urinary IGFBP-7 and TIMP-2, particularly when combined, are valuable early biomarkers for detecting CA-AKI in children. A prospective, single-center clinical trial included 172 children aged 0–18 years who received intravascular injections of a contrast medium. CA-AKI occurred in 20 out of 137 patients (14.6%). Urinary levels of NGAL, IGFBP-7, TIMP-2, and [IGFBP-7] × [TIMP-2] were significantly elevated in the CA-AKI group at 2 and 6 h post-contrast medium injection. Specifically, the combination of IGFBP-7 and TIMP-2 at a cutoff value of 0.417 (ng/mL)^2^/1000 demonstrated the best diagnostic performance with a specificity of 80.0% and a sensitivity of 81.2%. The AUC-ROC for [IGFBP-7] × [TIMP-2] was 0.811 (95% CI: 0.681–0.941), indicating superior predictive value compared to individual biomarkers [78].

## 5. Clinical Application

The clinical application of TIMP-2 has gained significant attention, particularly in the context of AKI. TIMP-2, which is typically tested in conjunction with IGFBP-7, has become a critical biomarker for kidney injury prediction and the provision of clinical care guidelines. NephroCheck^®^ is a test that is developed in conjunction with TIMP-2 and IGFBP-7 to help predict AKI early. This test examines the levels of TIMP-2 and IGFBP-7 in the urine and generates an AKI risk score. This combination of biomarkers is helpful in identifying patients who are at high risk of developing AKI in critical care settings, such as ICUs and post-surgery scenarios [79,80]. Numerous clinical investigations have confirmed the excellent sensitivity and specificity of TIMP-2 and IGFBP-7 in predicting AKI [79]. Guzzi et al. (2019) conducted a comprehensive analysis with a panel of expert clinicians to emphasize the clinical value of the biomarkers TIMP-2 and IGFBP-7. The [TIMP-2] × [IGFBP-7] test could be useful in patients undergoing major surgery, who are hemodynamically unstable, or have sepsis. It should be performed during perioperative treatment for patients undergoing major surgeries and identifies people at high risk of AKI, allowing for prompt actions such as optimizing hemodynamic status, avoiding nephrotoxic exposures, and implementing renal protective measures. [TIMP-2] × [IGFBP-7] testing is beneficial in hemodynamically unstable people, including those experiencing shock or severe cardiac events [80]. Measurement of TIMP-2 and IGFBP-7 can also be useful in both pediatric and neonatal populations. These indicators predict AKI in infants and children following cardiac surgery [72]. The degree and duration of kidney impairment can be predicted together with the start of AKI. For example, elevated levels of TIMP-2 and IGFBP-7 are linked to extended delayed graft function in kidney transplant recipients, which helps determine patient prognosis and graft viability [81]. These biomarkers can also aid in the distinction between acute and chronic AKI, influencing therapy choices and enhancing patient care [82].

## 6. Discussion

Numerous intriguing candidates have been identified in the search for accurate biomarkers in AKI. Each has pros and cons of their own. Important information on the relative effectiveness of TIMP-2 and potential use in clinical settings can be gained by comparing it to other well-established biomarkers, such as kidney injury molecule-1 (KIM-1), neutrophil gelatinase-associated lipocalin (NGAL), and interleukin-18 (IL-18) (Table 2) [83].

The roles of NGAL, KIM-1, and IL-18 in the early identification of AKI have been well studied. When kidney injury occurs, the protein NGAL, which is produced in neutrophils and different epithelial tissues, is increased, indicating tubular damage. As a hallmark of epithelial cell injury, KIM-1 is expressed in injured proximal tubule cells, while IL-18 is a pro-inflammatory cytokine that plays a role in the inflammatory response after AKI. These biomarkers have limitations even if they offer useful information [84]. For instance, NGAL can be elevated in other inflammatory conditions such as infections, trauma, or systemic inflammation, not exclusively due to kidney injury, which reduces its specificity for AKI and can lead to false positives [83,85]. Similarly, the expression of KIM-1 can be influenced by chronic kidney diseases, making it challenging to distinguish between acute and chronic conditions [86]. IL-18 levels can be affected by various inflammatory diseases, reducing its specificity for AKI [84,87].

TIMP-2, especially when combined with IGFBP-7, has demonstrated a very good diagnostic accuracy for AKI. In a study of high-risk surgical patients, [TIMP-2] × [IGFBP-7] presented an outstanding predictive performance with an AUC of 0.85 [50]. This level of accuracy exceeds those of NGAL and KIM-1, which have AUC values ranging from 0.70 to 0.80. TIMP-2 and IGFBP-7 appear to have a higher sensitivity and specificity than established biomarkers. In critically ill patients, a [TIMP-2] × [IGFBP-7] threshold of 0.3 (ng/mL)^2^/1000 demonstrated a 92% sensitivity and a 72% specificity, beating NGAL and KIM-1 alone [61].

TIMP-2 and IGFBP-7 also have long-term effects such as the prediction of CKD and mortality in addition to being able to anticipate the onset of AKI. Higher risk of developing CKD and higher death rates are associated with elevated levels of [TIMP-2] × [IGFBP-7] [55]. However, the degree of this correlation varies according to the demographics of the patients and the underlying etiology of AKI [79]. The improved performance of TIMP-2 and IGFBP-7 can be attributed to their roles in cell cycle regulation. These biomarkers indicate cellular stress and G1 cell cycle arrest, which are early signs of kidney injury. This contrasts with biomarkers like NGAL and KIM-1, which largely show damage that has already happened [80].

The biological variability of TIMP-2 can be an obstacle to its use as a clinical biomarker [9]. The effectiveness of TIMP-2 in predicting AKI in critically sick pediatric patients may differ from that in adults [64]. During AKI, TIMP-2 and IGFBP-7 produce cell cycle inhibitors, resulting in cell cycle arrest. Variations in cellular stress and inhibitor expression among different patient populations may affect TIMP-2 levels and accuracy. Therefore, it is essential to standardize testing protocols and validate TIMP-2 across a wide range of patient demographics [88].

Urine output and the presence of comorbid conditions (CKD, diabetes mellitus, heart failure) may affect the predictive accuracy of TIMP-2. However, there is an ongoing debate on this matter. Urine output is often decreased in patients with AKI, which could have an impact on TIMP-2 levels. Conversely, elevated TIMP-2 levels have been linked to higher mortality rates and the need for RRT. Additionally, sepsis and other disorders can increase TIMP-2 levels, and this can undermine its ability to accurately detect kidney injury [89]. Severe illness and other inflammatory diseases also have a considerable effect on IGFBP-7 levels [90]. There are differing opinions on the best time to measure TIMP-2 and the threshold values. The lack of proven methods for applying TIMP-2 findings in clinical practice is another issue. Contradictory results may arise from differences in assay procedures, handling methods, and sample collecting timeframes. Standardized guidelines and techniques are required to ensure the reproducibility and comparability of TIMP-2 readings across diverse clinical settings.

## 7. Conclusions

TIMP-2 is a multidimensional regulator of kidney injury and repair, especially in the context of AKI. While TIMP-2, particularly when combined with IGFBP-7, has shown significant potential as an early diagnostic for AKI, its clinical application is not without obstacles. TIMP-2 can be detected and quantified using a variety of analytical methods, each having advantages and limits. ELISA has great sensitivity and specificity with outstanding reproducibility, zymography provides qualitative insights into protease inhibition, RT-PCR enables gene expression analysis, and SPR provides precise kinetic data on molecular interactions. The method used is determined by the specific needs of the study, such as sensitivity, specificity, and the nature of the sample being studied. It is challenging to use TIMP-2 as an independent marker because of its biological variability, context-specific responses, and effect due to co-morbid diseases. Different patient populations have different levels of diagnostic accuracy and predictive values; hence, using a variety of biomarker approaches is necessary to increase reliability. A variety of practical implementation challenges, such as the need for specific instruments and procedures, prohibit TIMP-2 testing from becoming extensively employed in ordinary clinical practice. In the future, diagnosing AKI will necessitate the creation of multi-biomarker panels that may include newly found biomarkers, such as TIMP-2. To be effective in clinical practice, these panels must be validated and standardized across multiple patient populations. To address some of the practical obstacles of introducing TIMP-2 testing, advances in diagnostic technologies are being investigated. Point-of-care testing devices and automated platforms for assessing TIMP-2 and other biomarkers are being developed to aid in the rapid and reliable assessment of kidney function in clinical settings. These technologies seek to give quick data that can inform immediate therapeutic decisions, particularly in emergencies and ICUs, where the early identification of AKI is critical [60]. Furthermore, additional research into the molecular underpinnings of TIMP-2, as well as its interactions with other biomarkers and clinical factors, is required. Although TIMP-2 is a marker for cell cycle arrest, which can suggest kidney damage early on, its precise role in the pathogenesis of AKI remains uncertain. TIMP-2 causes both inflammation and death in kidney cells, showing that it may operate as both an injury signal and an agent in the damage process [40]. This dual role poses a hurdle to the development of TIMP-2-targeted medications because blocking TIMP-2 may aggravate other disease processes.

## Figures and Tables

**Table 1:** Clinical evidence on TIMP-2 as a biomarker for acute kidney injury.

AKI	Study	Key Findings	Ref
AKI	Meta-analysis of 20 trials involving 3625 patients	Pooled sensitivity: 0.79; specificity: 0.70; AUC: 0.81.	[54]
Surgical Patients	Study with 107 patients post-surgery	[TIMP-2] × [IGFBP-7] showed a good predictive value for AKI with an AUC of 0.85.	[33]
Cardiac Arrest	Prospective multicenter study with 115 patients post-cardiac arrest	[TIMP-2] × [IGFBP-7] had strong discriminative capacity with an AUC of 0.91 for severe AKI.	[54]
Cardiac Surgery	Cohort study of 557 patients	Combined TIMP-2 and IGFBP-7 showed high predictive accuracy for AKI with an AUC of 0.70.	[58]
SA-AKI	Prospective observational study with 198 patients	Combined TIMP-2 and IGFBP-7 had an AUC of 0.782 for non-recovery of kidney function, improved to 0.822 with a clinical model.	[59]
Critically Ill Patients	Multicenter trial with 420 patients in ICUs	High sensitivity (92%) and improved predictive power with [TIMP-2] × [IGFBP-7] compared to clinical variables alone.	[60]
Pediatric AKI	Prospective cohort of 42 critically ill neonates	Elevated urine TIMP-2 levels linked to AKI, preceding traditional biomarkers.	[61]
Patients with COVID-19	Multicenter observational study with 300 patients with COVID-19-related ARDS	High predictive value for AKI at enrollment with an AUC-ROC of 0.89.	[62]
Drug-induced AKI	Study with 21 patients with AKI and 28 controls receiving nephrotoxic drugs	Significantly higher TIMP-2 and IGFBP-7 levels 2–3 days before AKI onset.	[63]
CA-AKI	Prospective trial with 172 children post-contrast medium injection	Elevated [TIMP-2] × [IGFBP-7] levels significant for predicting AKI with an AUC-ROC of 0.811.	[64]

Abbreviations: AKI, acute kidney injury; AUC: area under the curve; TIMP-2, tissue inhibitor of metalloproteinases-2; IGFBP-7; insulin-like growth factor binding protein 7; SA-AKI, sepsis-associated acute kidney injury; ICU, intensive care unit; ROC, receiver operating curve; ARDS, acute respiratory distress syndrome; COVID-19, Coronavirus Disease 19; and CA-AKI, contrast-associated acute kidney injury.

**Table 2:** Comparison of urinary TIMP-2 with other urinary AKI biomarkers.

Biomarker	Mechanism	Advantages	Disadvantages	Ref
TIMP-2	Inhibits MMPs, involved in ECM turnover and cell cycle arrest	Early detection of AKI, reflects cellular stress before significant damage	Biological variability, affected by underlying conditions	[55,59,63,64,70]
NGAL	Expressed in neutrophils, upregulated in response to kidney injury	Rapid increase post-injury	Reflects injury post-damage, not early predictive	[54,61]
KIM-1	Expressed in damaged proximal tubule cells	Specific for epithelial cell injury	Late indicator, reflects post-injury	[57,62]
IL-18	Pro-inflammatory cytokine involved in inflammatory response	Useful in inflammatory AKI	Less specific for kidney injury	[56,69]

Abbreviations: TIMP-2, tissue inhibitor of metalloproteinases-2; NGAL, neutrophil gelatinase-associated lipocalin; KIM-1, kidney injury molecule-1; IL-18, interleukin-18; MMPs, metalloproteinases; ECM, extracellular matrix; AKI, acute kidney injury.
